# Detecting epileptic seizures with electroencephalogram via a context-learning model

**DOI:** 10.1186/s12911-016-0310-7

**Published:** 2016-07-21

**Authors:** Guangxu Xun, Xiaowei Jia, Aidong Zhang

**Affiliations:** Department of Computer Science and Engineering, SUNY at Buffalo, Buffalo, USA

**Keywords:** Context learning, Epileptic seizure, Electroencephalogram, Feature extraction

## Abstract

**Background:**

Epileptic seizure is a serious health problem in the world and there is a huge population suffering from it every year. If an algorithm could automatically detect seizures and deliver the patient therapy or notify the hospital, that would be of great assistance. Analyzing the scalp electroencephalogram (EEG) is the most common way to detect the onset of a seizure. In this paper, we proposed the context-learning based EEG analysis for seizure detection (Context-EEG) algorithm.

**Methods:**

The proposed method aims at extracting both the hidden inherent features within EEG fragments and the temporal features from EEG contexts. First, we segment the EEG signals into EEG fragments of fixed length. Second, we learn the hidden inherent features from each fragment with a sparse auto-encoder and thus the dimensionality of the original data is reduced. Third, we translate each EEG fragment to an EEG word so that a continuous EEG signal is converted to a sequence of EEG words. Fourth, by analyzing the context information of EEG words, we learn the temporal features for EEG signals. And finally, we concatenate the hidden features and the temporal features together to train a binary classifier which can be used to detect the onset of an epileptic sezure.

**Results:**

4302 EEG fragments from four different patients are used to train and test our model. An error rate of 22.93 % is achieved by our model as a general, non-patient specific seizure detector. The error rate of our model is averagely 16.7 % lower than the other baseline models. Receiver operating characteristics (ROC curve) and area under curve (AUC) confirm the effectiveness of our model. Furthermore, we discuss the extracted features and how to reconstruct the original data based on the extracted features, as well as the parameter sensitivity.

**Conclusions:**

Given a EEG fragment, by extracting high-quality features (the hidden inherent features and temporal features) from the EEG signals, our Context-EEG model is able to detect the onset of a seizure with high accuracy in real time.

## Background

An epileptic seizure is a transient aberration due to abnormally excessive or synchronous neuronal activities in the brain. The disease epilepsy is defined as an enduring predisposition in brain which generates epileptic seizures. The symptom of epilepsy can vary from uncontrolled jerking movement to as subtle as a temporary unconsciousness [1]. Frequent seizures are dangerous as it may result in serious physical injuries and even death. According to the studies [2, 3], 5–10 % of the population over 80 years old have experienced the epileptic seizures for at least once. After the first experience, they would suffer from another epileptic seizure with a probability of 40–50 %. Currently about 1 % of the global population are affected by epileptic seizures and at some point in time the number used to be 4 % at its highest [2].

Considering the serious outcome caused by epileptic seizure for the patients and the large population affected by epileptic seizure, a device that can quickly detect the onset of seizure and deliver therapy can be of great help. In recent years, the surge in brain-computer interface (BCI) technology introduces tremendous opportunities to applying physiological signals to biomedical applications. According to previous studies, the electroencephalogram signals (EEG) are closely related to brain activities and can be used to detect neural diseases [4, 5]. Therefore, analysis on the EEG signals is a powerful and enabling way to detect the onset of seizures. While we can collect EEG signals from different parts of body, the scalp EEG is most widely adopted, which is a non-invasive, multi-channel recording of the brain’s electrical activities.

The condition of seizures has proven to be closely related to the neural electrical activities which are reflected on the scalp EEG signals. Nevertheless, there still exist several challenges in the automatic seizure detection task:

First, seizure and non-seizure states have considerable overlap in patients’ EEG signal patterns.

Second, both the seizure and non-seizure status have more than one sub-states and the EEG of an patient may constantly transition between them.

Third, most conventional learning models directly send all the inputs to the seizure classifier without extracting features and are not able to analyze the correlation between different input data, which would result in the failure to recognize the temporal signal patterns [6–8].

Fourth, the characteristics of seizures on EEG might vary significantly across patients. Because of this cross-patient variability in seizure and non-seizure activities, patient non-specific classifiers are usually not able to obtain a high accuracy and suffer from long delays in detecting the onset of a seizure. On the contrary, patient specific classifiers can exhibit impressive performance but they are not able to work for new patients [9].

Fifth, in practice, the automatic seizure detector should be able to detect the onset of a seizure quickly.

Sixth, practically, it should also be able to handle the unbalanced training data, because seizures are rare events which results in the paucity of seizure training data.

Facing these challenges, we propose an innovative method to capture the temporal features and context information hidden in EEG data. Since the onset of a seizure is related to a sequence of EEG signals rather than the values at a certain point, temporal analysis is necessary and crucial for the seizure detection. The context-learning based techniques have proven to be effective in bioinformatics [10, 11]. More specifically, our model handles these challenges with the following strategies:

- We segment the EEG into small pieces of fixed length with a sliding window. By sliding the window with a fixed step length, the EEG is segmented into numerous small fragments as the ‘EEG words’ for further analysis.
- We extract the hidden inherent features within each EEG fragment. One single feature corresponds to one ‘EEG word’ in our learned ‘EEG dictionary’.
- We explore the temporal knowledge by learning the context information of EEG fragments. After translating the EEG fragments into ‘EEG words’, we can infer the context knowledge for each EEG fragment by analyzing it with its context fragments.
- Finally, we combine the hidden features of each EEG fragment and the temporal knowledge together and subsequently send them to a seizure classifier. In this paper we propose a two-way classifier (seizure or non-seizure states) while the proposed model can be easily extended to uncover more physiological classes.

**Fig. 1:**
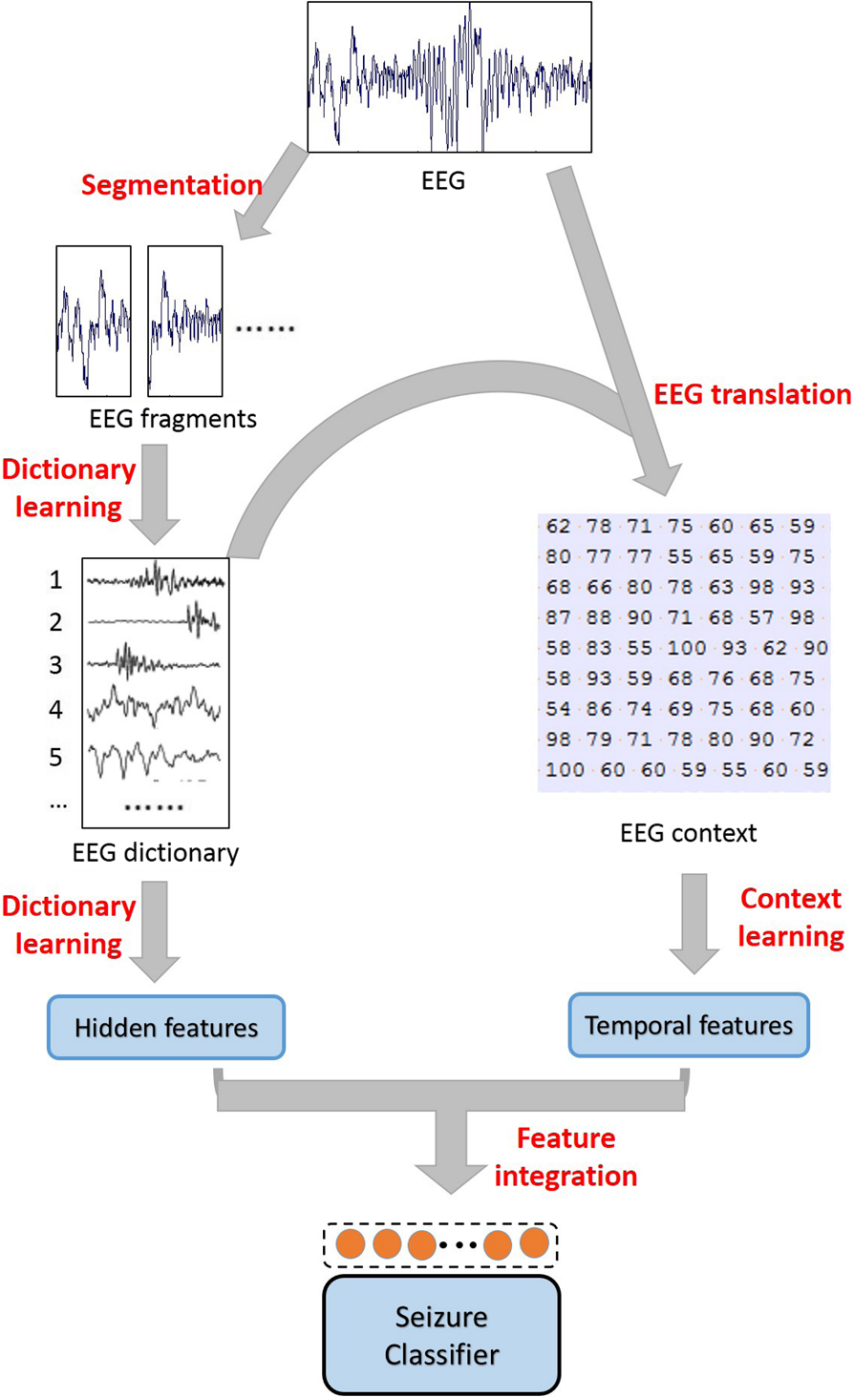
Schematic illustration of the overall framework. In this framework, we focus on extracting representative features from EEG signals. On one hand we extract the hidden inherent features of each EEG fragment by segmenting the EEG signals and learning the EEG dictionary. On the other hand, we extract the temporal features by analyzing the EEG contexts of EEG fragments. And finally, we send the integrated features to the classifier

Based on the above strategies, the framework of our model is described in Fig. 1.

## Methods

### Dataset

The dataset we use is the scalp electroencephalogram collected at the Children’s Hospital Boston [5]. EEG measures the electrical activities in the brain by attaching multiple electrodes to the patient’s scalp. Each EEG channel records the voltage change between a specific pair of electrodes, and therefore reflects the electrical activities in the corresponding region. This study used public EEG signal obtained from the CHB-MIT scalp EEG database on PhysioNet. A team of investigators from Children’s Hospital Boston (CHB) and the Massachusetts Institute of Technology (MIT) created and contributed this database to PhysioNet. The CHB Internal Review Board approved the study under the overall CHB-MIT scalp EEG project.

This dataset consists of the EEG recording intractable seizures from pediatric subjects. Twenty three patients were involved in the dataset, including 5 males and 18 females from age 2 to age 22. In order to characterize their seizures and assess the necessity of surgery for them, their scalp EEG signals were recorded. All the signals were recorded at 256 *Hz* with 16−*bit* resolution. In most files, there are 23 EEG channels and 24 channels in a few cases.

**Fig. 2:**
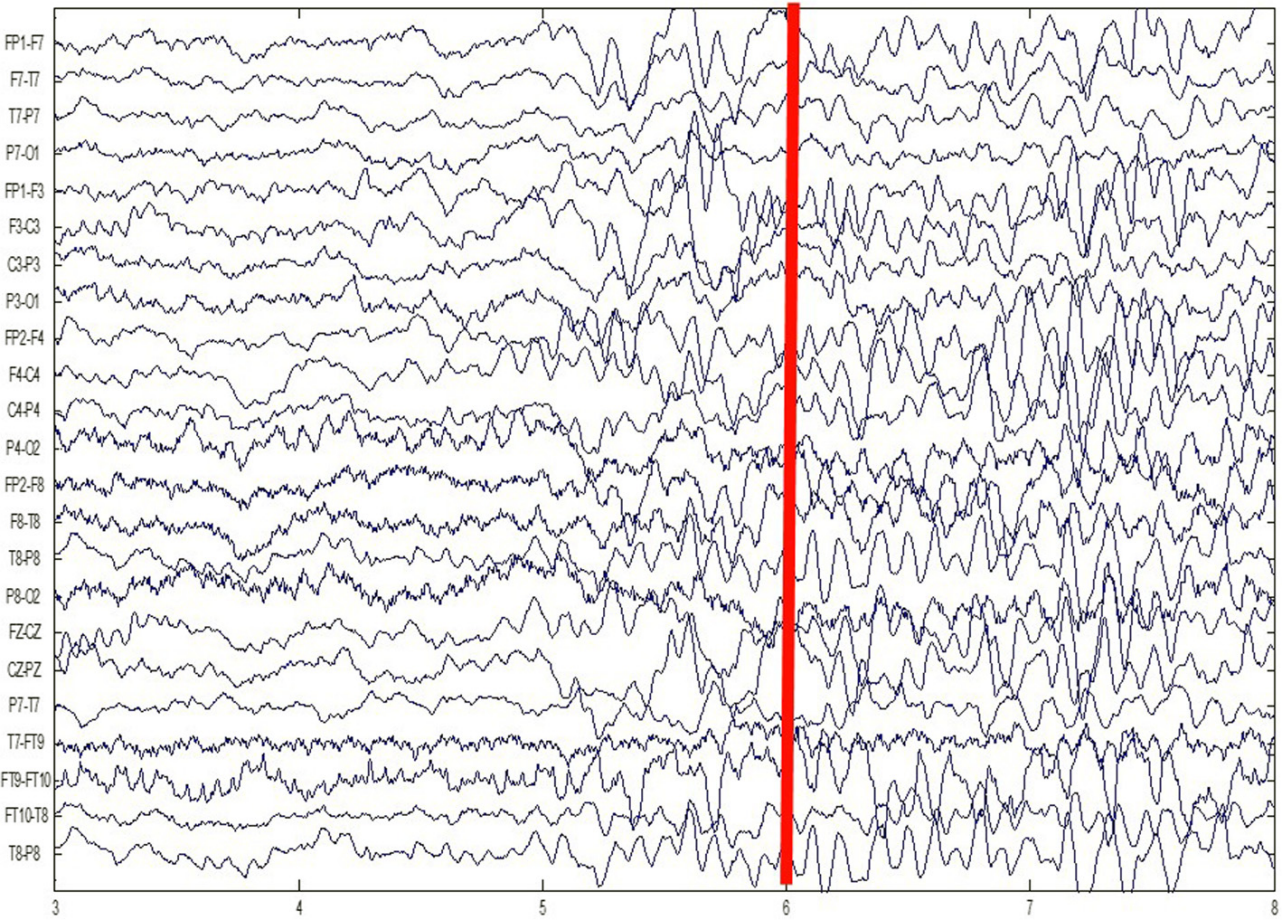
The scalp EEG of patient A. The red bar marks the onset of a seizure. The EEG signals before this red bar are of non-seizure states and the EEG signals after this red bar are of seizure states

Usually following the onset of a seizure, a set of EEG signals show dramatic changes from the non-seizure states. And this will assist the seizure detector in distinguishing the seizure and non-seizure states. For example, Fig. 2 illustrates the onset of a seizure of patient A. Patient A’s seizure starts at the 6th second as the red bar shows in Fig. 2, and then the onset of this seizure comes with the significant changes of EEG signals.

**Fig. 3:**
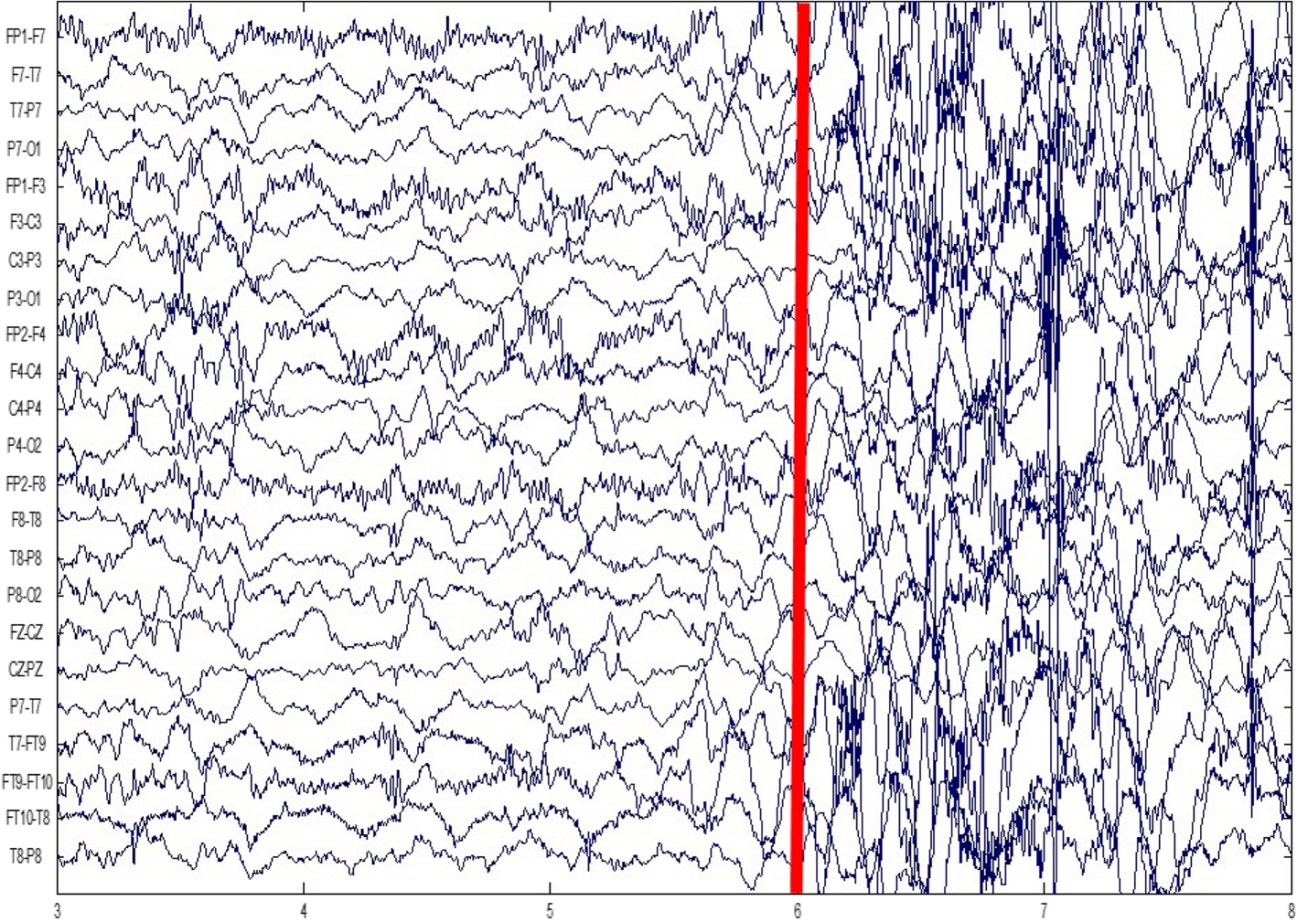
The scalp EEG of patient B. The red bar marks the onset of a seizure. The EEG signals before this red bar are of non-seizure states and the EEG signals after this red bar are of seizure states

However, as we mentioned, the characteristics of seizures on EEG might vary significantly across patients, and this variability will make the seizure detection problem even more difficult. Figure 3 shows the onset of a seizure of patient B. Patient B’s seizure also starts at the 6th second as the red bar shows in Fig. 3. Significant changes can still be observed between seizure and non-seizure states, but the pattern of seizure EEG differs across patients.

Moreover, sometimes EEG signals show certain kinds of rhythmic activities when people are excited, which are also different from the calm states. But these EEG fragments should not be confused with seizures. The ambiguity brought by these activities requires the seizure detector to learn the features of seizures.

### The Context-EEG model

In this subsection, we propose a novel framework to extract the hidden inherent features and temporal information in EEG signals. We start by discussing the first step of our model, segmenting EEG data.

#### EEG segmentation

Since EEG signals cannot be explicitly segmented into sub-fragments associated with physiological meanings, we segment it into several overlapped epochs of fixed length. With a sliding window of length *L* (for example, 3 seconds), we build our EEG fragment pool by sliding the window by 1 second at each step.

**Fig. 4:**
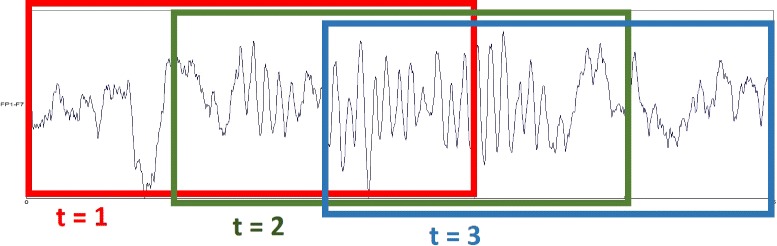
An example of EEG segmentation. Given a 5 second long EEG signal, it is segmented into three EEG fragments by a 3 second sliding window when the step length is 1 second

Figure 4 shows an example of how to segment an EEG signal into three fragments, in which the length of the sliding window is fixed to *L*=3 seconds and the step length is 1 second.

By segmenting the EEG signals into numerous EEG fragments, we obtain an EEG pool of EEG fragments. Our further analysis and experiments are conducted on these EEG fragments.

#### EEG dictionary learning

After the EEG fragment pool is built, we use a sparse autoencoder (SAE) to extract features for these fragments. Autoencoder is an unsupervised neural network based model which aims at discovering interesting structures of data by reconstructing the input [12].

In its simplest form, an autoencoder consists of two parts, an encoder and a decoder. Autoencoder can also be viewed as a technique for feature extraction and dimensionality reduction. The encoder reduces the dimensionality of the input data to obtain principal features, and the decoder is tuned to reconstruct the input data based on the output features of the encoder. Hence by minimizing the reconstruction error, we can get the optimal autoencoder whose encoder extracts features with reduced dimensionality and decoder reconstructs input data from the extracted features. In particular, when we select a linear activation function and use less hidden units than the input dimensions, the encoder works similarly with the principal component analysis (PCA) [13]. However, when a non-linear activation function is adopted, the autoencoder has proven to be capable of learning more useful features than PCA [14].

**Fig. 5:**
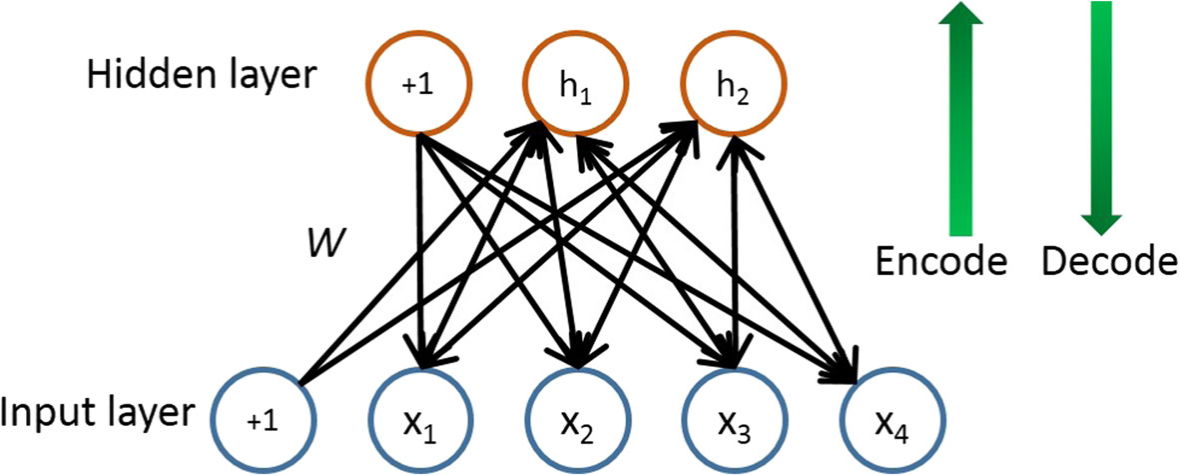
The structure of an autoencoder. *x* is the input data, *h* is the hidden unit and +1 term is adopted to integrate the bias. It is composed of two parts, an encoder and a decoder. The output of the encoder is the extracted features for the input data and the decoder is tuned to reconstruct the input data based on the extracted features

Figure 5 depicts the structure of an antoencoder with one hidden layer, where *x* is the input data, *h* is the hidden unit and +1 term is adopted to integrate the bias. The autoencoder tries to learn a hidden layer that satisfies *g*(*f*(*x*))≈*x*, where *f*(*x*) extracts features from input *s* and *g*(*y*) reconstructs the original data from the extracted features. In other words, it aims at learning a model to approximate the output with the input. The bottom-up structure denotes the process of encoding, as the left green arrow shows in Fig. 5. The encoder corresponds to the function *f* that maps the input data *x* to the hidden layer *h*. The function *f* is defined as:

1 $$ h=f(x)=\sigma (Wx+b_{h}),  $$

where *b*_*h*_ represents the bias, *W* represents the weight matrix from input data to the hidden units and *σ*(*x*) is the activation function. In our test we adopt the non-linear logistic sigmoid function, as follows:

$$\sigma (x)=\frac{1}{1+e^{-x}}. $$

The top-down structure denotes the process of decoding, as the right green arrow shows in Fig. 5. The decoder corresponds to the function *g* that reconstructs the input data from the hidden features:

2 $$ \tilde{x}=g(h)=\sigma '(W'h+b_{x}),  $$

where *b*_*x*_ is the bias, *W*^′^ is the weight matrix from the hidden features to the reconstruction and *σ*^′^(*x*) is the activation function of the decoder. Usually the decoder adopts the same activation as the encoder for simplicity, and thus for the decoder we adopt the logistic sigmoid function as well.

As the autoencoder aims at reconstructing the input data, the cost function in terms of parameters *θ*={*W,W*^′^,*b*_*h*_,*b*_*x*_} is defined as:

3 $$ J_{AE}(\theta)=\sum_{x}L(x,\tilde{x})=\sum_{x}L(x,g(f(x))),   $$

where *L* represents the reconstruction error, which is measured by the cross-entropy loss:

$$L(x,\tilde{x})=-\sum_{x}(x\log (\tilde{x})+(1-x)log(1-\tilde{x})). $$

In our case, given an 3-second EEG fragment, the input size for the autoencoder is 256 *Hz*×3 *seconds*=768. This may result in the number of hidden units being also very large. For each input EEG fragment, intuitively it should only activate a few of the features rather than most of the features. So when the number of hidden units is large, we can still discover some interesting structure by imposing a sparsity constraint on the hidden units [15]. Thus, we use a sparse autoencoder to deal with our EEG dictionary learning task.

A hidden unit is considered of being ‘inactive’ when its output is close to zero, and of being ‘active’ when its output is close to one. Specifically the sparsity constraint makes one hidden unit inactive most of the times. For hidden unit *j*, we define its average activation $\hat {\rho _{j}}$ over all the input data *x* as:

$$\hat{\rho_{j}}=\frac{1}{N}\sum_{i=1}^{N}(f_{j}(x_{i})), $$

where *N* is the size of input data, and *f*_*j*_(*x*_*i*_) is the output of hidden unit *j* on the *i*-th input data. Then the average activation is constrained to the sparsity parameter *ρ* which should be quite small (for example *ρ*=0.05). To measure the difference between *ρ* and $\hat {\rho _{j}}$, Kullback-Leibler (KL) divergence is adopted [16]:

$$KL(\rho\|\hat{\rho_{j}})=\rho\log\frac{\rho}{\hat{\rho_{j}}}+(1-\rho)\log\frac{1-\rho}{1-\hat{\rho_{j}}}. $$

This function penalizes the hidden units being active for too many times. Incorporating the sparsity constraint into the autoencoder cost function in Eq. 3, we get the overall cost function for the sparse autoencoder:

4 $$ J_{SAE}(\theta)=J_{AE}(\theta)+\beta\sum_{j=1}^{M}KL(\rho\|\hat{\rho_{j}}),  $$

where *J*_*AE*_ is the cost function defined in Eq. 3, *β* is the parameter that controls the weight of the sparsity constraint and *M* is the number of hidden units. It is noteworthy that, the average activation $\hat {\rho _{j}}$ is also a function of *θ*. We use backpropagation to update the parameters, and the gradient of the cost function is computed as:

5 $$ \frac{\partial J_{SAE}}{\partial w(k)}=\frac{\partial J_{AE}}{\partial w(k)}+\beta\left(-\frac{\rho}{\hat{\rho_{k}}}+\frac{1-\rho}{1-\hat{\rho_{k}}}\right).  $$

The sparse autoencoder we have discussed above has only one hidden layer, and in our model, we use a sparse autoencoder with two hidden layers, which is able to capture more abstract features. Each learned feature corresponds to a vocabulary in the EEG dictionary. And the EEG dictionary is constructed by decoding all the learned features.

#### EEG sequence translation

As the sparse autoencoder is trained, each EEG word is obtained by decoding each hidden unit and one EEG word represents one basic signal type. The EEG dictionary is a set of all the EEG words, and each EEG fragment can be viewed as a combination of EEG words in the dictionary. For each EEG fragment, different features have different weights due to the different proportions of basic signal types in it. Therefore the EEG fragment can be sampled to a single EEG word according to the normalized feature weights.

Given a continuous EEG signal, we can translate it into a sequence of words by converting each EEG fragment of it into an EEG word in the dicionary. Translating continuous signal into discrete words would help us learn the temporal context information in further analysis.

**Fig. 6:**
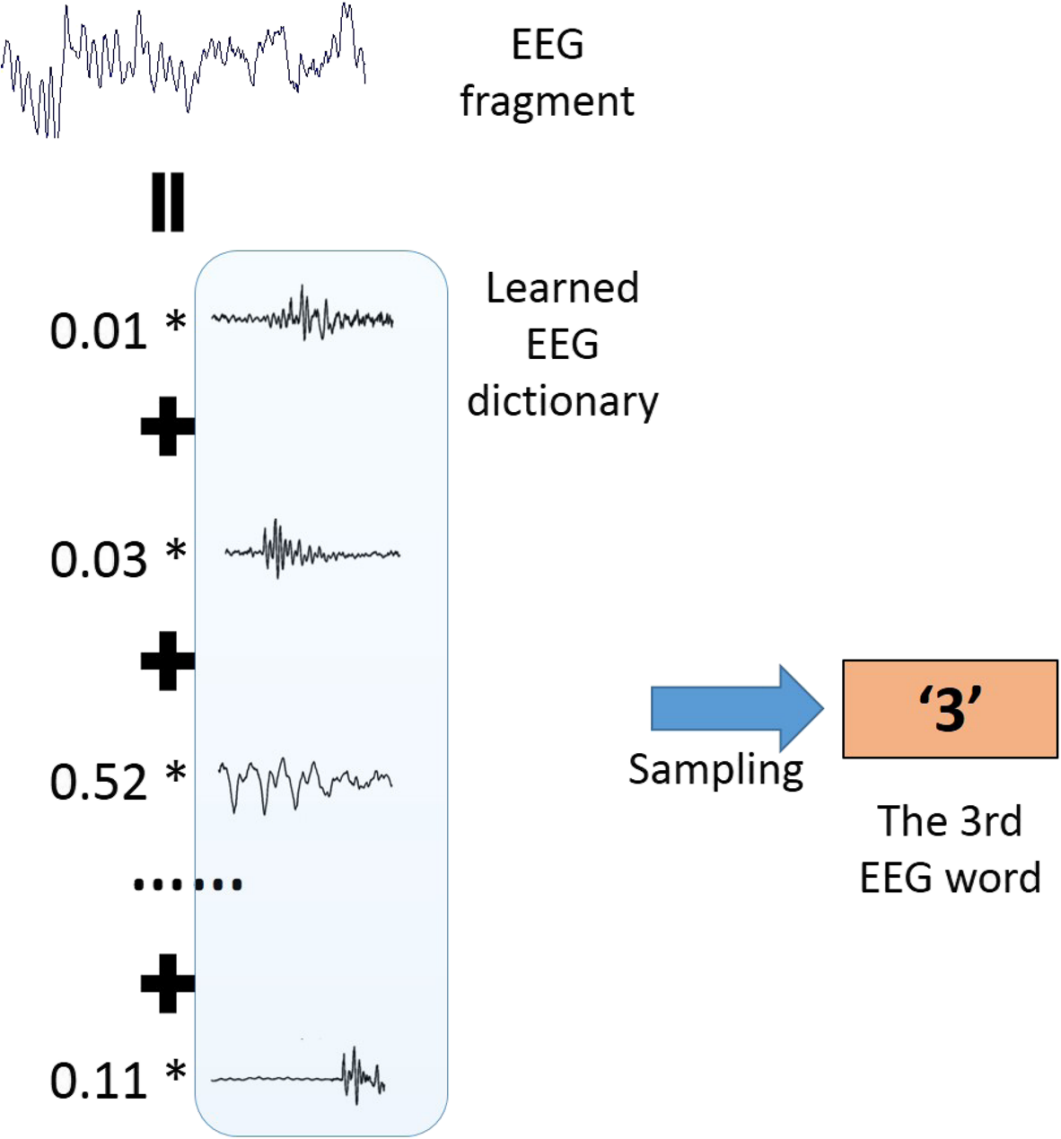
An example of EEG sequence translation. Each EEG fragment can be decomposed into a combination of EEG words in the EEG dictionary. Each EEG word corresponds to a hidden unit of the sparse autoencoder in the dictionary learning step, and the distribution of EEG words is the normalized weights of the hidden units. And then an EEG fragment is sampled to an EEG word according the word distribution

Figure 6 shows an example of how to translate an EEG fragment to the corresponding EEG word based on the learned dictionary. More specifically, given an EEG fragment and the EEG dictionary learned by the sparse autoencoder, the EEG word in the dictionary corresponding to this EEG fragment is drawn from the multinomial distribution:

$$P(\epsilon_{i})=\frac{h_{i}}{\sum_{j=1}^{M}h_{j}}, $$

where *ε*_*i*_ is the *i*-th EEG word in the dictionary, *h*_*i*_ is the output of the *i*-th hidden unit on this EEG fragment input and *M* is the number of hidden units. Because of the sparsity constraint, most of the *h*_*i*_ should be close to zero, which means each EEG fragment is basically composed of a few main signal types.

#### EEG context learning

In order to capture the temporal features of seizures on EEG signals, we design an EEG context learning algorithm to analyze the EEG sentences.

In the previous EEG translation step, every EEG fragment is translated to an EEG word, so the continuous EEG signals are translated to EEG sentences of EEG words. In this way, we are able to learn the temporal features hidden in the context information of the EEG sentences.

The main idea of the EEG context learning algorithm is to infer the current EEG word based on its context words. This intuition is inspired by the Continuous Bag-of-words (CBOW) model [17], where each word is represented by a vector of fixed length and words with similar semantics would be mapped to close positions in the vector space by learning the context information.

In our model, the context of an EEG word is drawn from the EEG sentence with a window of length 2*k*+1, i.e., the previous *k* words and the following *k* words form the context of the current word.

**Fig. 7:**
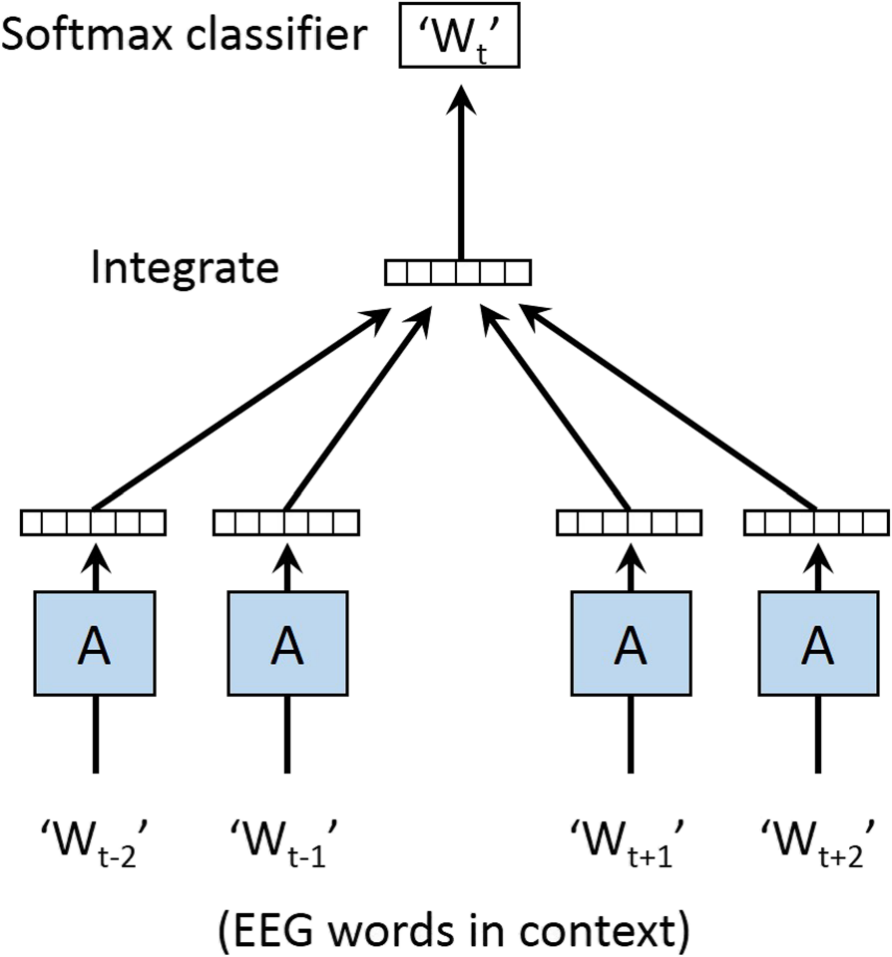
The framework of the EEG context learning algorithm. This framework adopts a neural network structure to extract the temporal features from EEG contexts. The input layer is a projection layer that projects each word to an unique vector. The hidden layer is an integration of the vectors in a context. And the output layer is a classifier where each tuple in the output corresponds to the conditional probability of a word in the dictionary

Figure 7 shows the framework of this EEG context learning algorithm. *W*_*t*_ is the current word to predict, and *W*_*t*−2_∼*W*_*t*+2_ are the context words of *W*_*t*_. Each EEG word is mapped to an unique vector, represented by a column in matrix *A*. The integration of all the word vectors in context should lead the softmax classifier to choose the current word *W*_*t*_.

More formally, our EEG sentence dataset consists of T training EEG words *a*_1_,*a*_2_,…,*a*_*T*_. We are going to predict each EEG word based on its neighborhood. So all the context EEG word vectors make a contribution to the prediction task about the current word in the context. Thus the objective of this EEG context learning algorithm is to maximize the average log probability:

6 $$ L=\frac{1}{T}\sum_{t=k}^{T-k}\log p(a_{t}|a_{t-k},a_{t-k+1},\ldots,a_{t+k}),   $$

where 2*k*+1 is the size of the context window, i.e., when predicting every EEG word, only its previous *k* words and following *k* words contribute to the prediction about this word as its context. The other EEG words outside this context window are not considered.

The *p*(*a*_*t*_|*a*_*t*−*k*_,*a*_*t*−*k*+1_,…,*a*_*t*+*k*_) in Eq. 6 is the prediction task for EEG word *a*_*t*_. It is calculated by a multi-class softmax classifier, as follows:

7 $$ p(a_{t}|a_{t-k},a_{t-k+1},\ldots,a_{t+k})=\frac{e^{y_{a_{t}}}}{\sum_{i}e^{y_{i}}},  $$

where *e*^*x*^ is the exponential function and *y*_*i*_ is the unnormalized log probability for each output EEG word *a*_*i*_. As Fig. 7 shows, the process of prediction is based on a two-layer neural network, which means there are three steps for each prediction: first we need to project all the input words into the vector space, and second we integrate the vectors, and finally we calculate the output *y*. So $y_{a_{t}}$ can be computed as:

8 $$ y_{a_{t}}=b+Uh(a_{t-k},a_{t-k+1},\ldots,a_{t+k};A),  $$

where *b* and *U* are the parameters of the softmax classifier, and *h* is the integration of the context EEG word vectors extracted from matrix *A*. The integration is typically implemented as either average function or the concatenation.

**Fig. 8:**
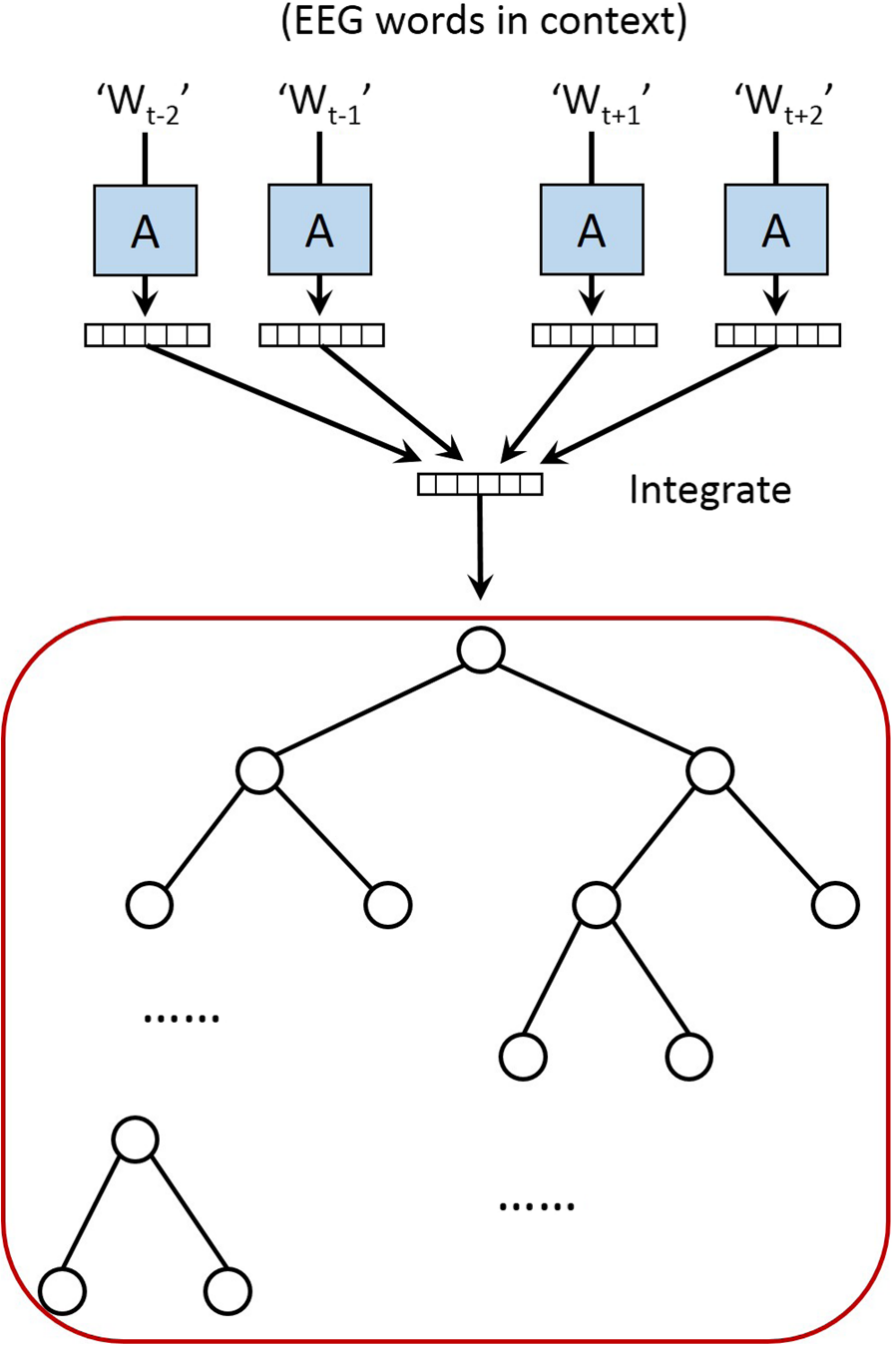
The illustration of the hierarchical softmax. The output layer is replaced by a hierarchical tree structure and each leaf node corresponds to one word in the vocabulary. So that the time complexity is drastically reduced. More specifically, a binary Huffman tree structure is adopted by our model, wherein the shortest paths are assigned to the most frequent words

In practice, for the sake of fast training, the softmax classifier is usually replaced by the hierarchical softmax classifier. In our model, the hierarchical softmax classifier is based on a binary Huffman tree as shown in Fig. 8, wherein the shortest path is assigned to the most frequent EEG word.

Applying hierarchical softmax classifier accelerates our model in three ways: first, according to the strategy of building the Huffman tree, frequent EEG words are assigned short codes, which means the overall accessing time is shorter; second, by representing the vocabulary with a binary tree structure, the average seeking time reduces from *O*(*N*) to *O*(log(*N*)), where *N* is the size of the vocabulary and log(*N*) is the height of the Huffman tree; third, by storing the EEG words in a tree structure, in each round of update, we only need to access and update the nodes on the path rather than accessing all the words in the vocabulary.

Similar with the other neural network models, the EEG context learning algorithm is trained with backpropagation. After learning EEG contexts, the EEG words with similar properties are mapped to close positions in the vector space [18]. These vectors can be used as the temporal features because in the EEG context learning process, the order of the EEG words in an EEG sentence is considered as part of the context information.

#### Epileptic seizure detection

The final features we use are the combination of the hidden inherent features within the EEG fragments extracted in the EEG dictionary learning process and the temporal features extracted in the EEG context learning process.

After concatenating the hidden inherent features and the temporal features, we use them together with the labels to train a support vector machine (SVM) classifier [19]. And we name our model the Context-Learning Based EEG Analysis for Seizure Detection (Context-EEG).

By incorporating the hidden inherent features and temporal features into the classifier, SVM is able to find a more distinct hyperplane between the seizure and the non-seizure EEG fragments.

## Results

We conduct computational experiments to show the effectiveness of our model Context-EEG for detecting the onset of an epileptic seizure. To achieve this, we benchmark our model on the CHB-MIT scalp EEG dataset mentioned in the ‘Background’ Section.

### Task and baselines

In order to design a general seizure detecting algorithm, i.e., a non-patient specific seizure detecting algorithm, we combine 4302 EEG fragments from four different patients as our experiment dataset, and randomly choose 3500 EEG fragments out of it as the training set and use the other EEG fragments as the test set. Given a piece of EEG fragment, it’s a two-way classification task where the class labels are *{seizure, non-seizure}*. We measure the performance of each algorithm by the classification error rate, the receiver operating characteristic (ROC curve) and the area under curve (AUC).

Since it is a classification task, we apply several widely used classification algorithms as the baseline algorithms, including SVM and neural network (NN) [20]. For the sake of fairness and to avoid the curse of dimensionality, it is necessary to reduce the dimensionality of the data before we send it to SVM and NN. So we employ the decimation process by downsampling the EEG signals, and we call the SVM with downsampling DSVM, the NN with downsampling DNN. Also, we use the principal component analysis (PCA) [21] as another data preprocessing mechanism to reduce the dimensionality of the data, and we call the SVM with PCA as PSVM, the NN with PCA as PNN. SVM is also adopted by our Context-EEG model as the classifier. The input to it is the hidden inherent features and the temporal features of each EEG fragment extracted by our model, and the dimensionality of the data is reduced as the features are extracted.

### Experiment protocols

In our dataset, a seizure is usually 30 ∼100 seconds long and surrounded by one hour long non-seizure signals, which means seizures are rare events. Considering the rarity of seizure events, we trim our test set to balance the number of seizure fragments and non-seizure fragments to around 50–50. Otherwise, simply by labeling all the test samples as non-seizure state, a classifier can obtain an error rate as low as 30*s*/3600*s*=0.8333 *%*.

Since the original sampling rate is 256 *Hz* and each file contains 23 channels of EEG signals, a 3-second long EEG fragment consists of 256 *Hz*×3 *seconds*×23 *channels*=17664 data points. This high dimensionality problem would not only put the classifiers at the risk of the curse of dimensionality, but also consume a lot of time and space. So for the baseline methods with decimation process, we reduce their dimensions of the input data to the same dimensions as Context-EEG by PCA and downsampling.

### Experimental result

The error rates of different methods are reported in Table 1. We can see that our model Context-EEG outperforms the other methods by averagely 16.7 %. It is worth noticing that the performances of SVM and NN decrease quite a lot as the dimensionality of their input data decreases. The original SVM performs well at the cost of acceptable dimensionality. However, after reducing its dimensionality to the same dimensionality as ours, PSVM and DSVM perform much worse. And the comparison result between two different decimation approaches also shows that using PCA is a better way to extract principal components of data than just simply downsampling.

**Table 1:** The error rates of each method

Methods	Error rate
SVM	23.43 %
NN	26.22 %
DSVM	29.71 %
DNN	30.21 %
PSVM	28.71 %
PNN	26.82 %
**Context-EEG**	**22.93 %**

Our model Context-EEG outperforms the baseline methods by 16.7 % on average

**Fig. 9:**
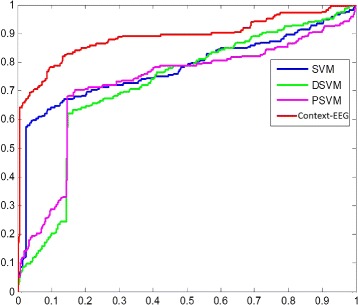
The ROC curves of the proposed model and the baselines. In this figure, the blue line is the ROC curve of SVM. The green ROC curve is for the DSVM and purple ROC is for the PSVM. The red curve is the ROC of Context-EEG

Figure 9 and Table 2 show the ROC curve and the AUC of each method respectively. We can see that our model performs much better than the other methods.

**Table 2:** The AUC of each method

Methods	AUC
SVM	0.7764
DSVM	0.7208
PSVM	0.7232
**Context-EEG**	**0.8880**

The AUC of our model is higher than the other methods by 14 % averagely

In the ROC curve figure, among all the baseline methods, SVM performs the best. However, even though the dimensionality of SVM is 64 times as high as the dimensionality of the Context-EEG model, SVM still performs much worse than the Context-EEG model. And we can see that, the true positive rate of the Context-EEG model increases at a very fast speed in the beginning when the false positive rate is still close to zero, which means Context-EEG is able to capture the important features to represent and separate seizure and non-seizure data points effectively.

As shown in Table 2, the AUC of the Context-EEG model is higher than the other methods’ AUCs by 14 percent averagely even though it has the lowest dimensionality among all the methods. As with the error rate results and the ROC curves, reducing the dimensionality of input data for SVM by downsampling and PCA has a rather big impact on the AUC, and using PCA to extract principal components is a little bit better than just simply downsampling the data.

It is worth noticing that the Context-EEG model is slow at learning but extremely fast at prediction, because once the training step is finished, the parameters will be stored and will not change anymore. When a new EEG fragment comes, the classification will be done in *O*(*N*) time. So as a real-time seizure detecting algorithm, Context-EEG would be of great help for the patients in practice.

## Discussion

The main achievement of this paper is an automatic, general, non-patient specific seizure detector which is able to extract both the hidden inherent features and the temporal features from EEG signals. The experimental results confirm the effectiveness of our model. Being able to extract high-quality features is the main reason why our model outperforms the baselines. Thus, in the following subsection we show the extracted features and how the original data can be reconstructed from the extracted features.

### EEG dictionary learning and EEG signal reconstruction

The first step of our feature extraction process is to learn the hidden inherent features within each EEG fragment. In this step, we learn the hidden inherent features by setting the output of the sparse autoencoder equal to the input. Hence we can claim that the features are well learned if the features are able to precisely reconstruct the input data. Because only if the learned features contain all the crucial information of the input data, we can reconstruct the data based on the learned features.

Usually the dimensionality of the features is much lower than the dimensionality of the original data, so as we are extracting the hidden inherent features of EEG fragments, we are also reducing the dimensionality.

**Fig. 10:**
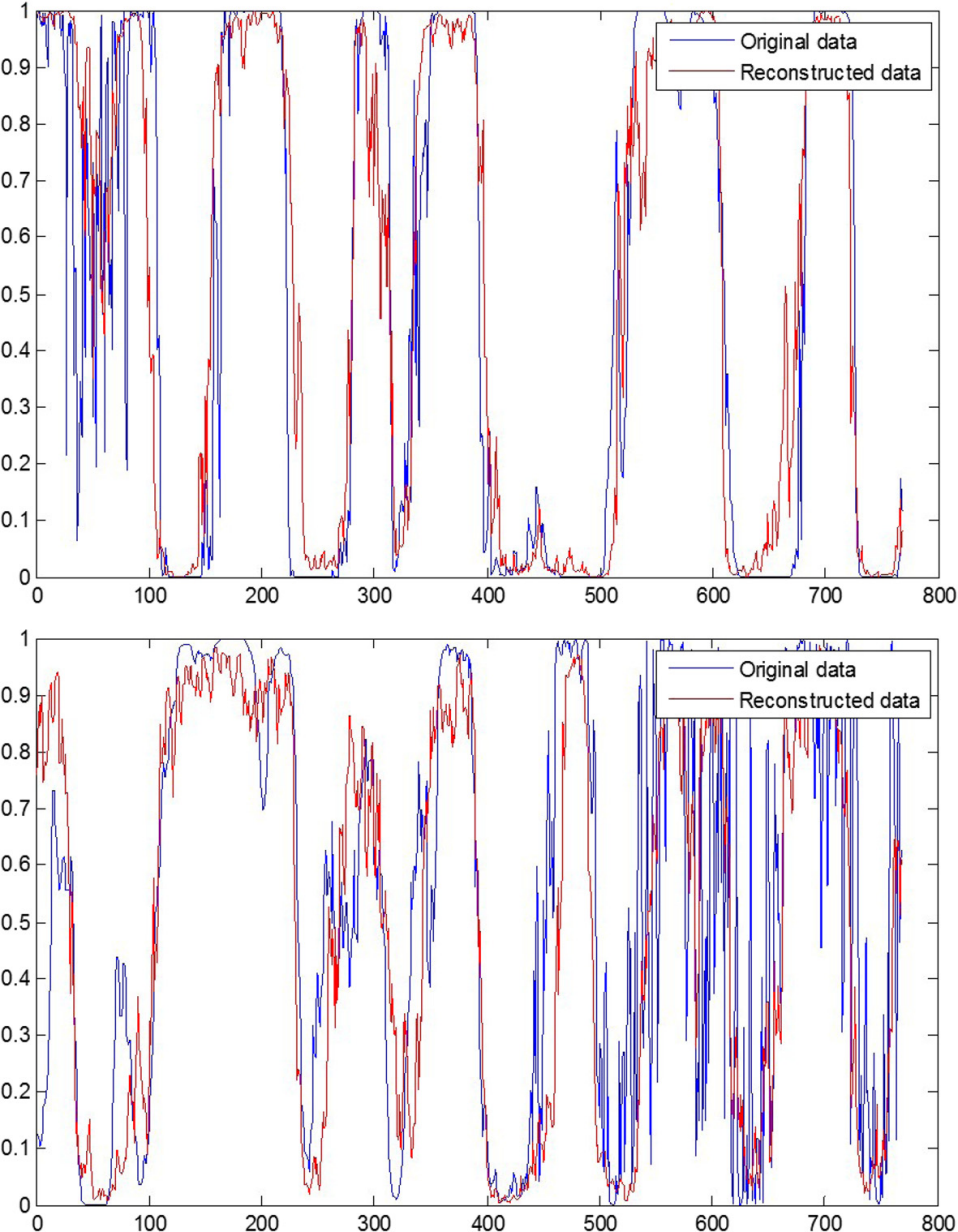
Two examples of EEG data reconstruction. The blue lines are the original data and the red lines are the data reconstructed by the hidden inherent features extracted in the EEG dictionary learning step

Figure 10 illustrates the EEG data reconstruction process. We can see that the reconstructed data (the red lines in the figure) is quite similar with the original data (the blue lines in the figure). The upper figure is an EEG fragment of a non-seizure state, where the EEG signal is regular and clean. And in this case, the learned features successfully reconstruct every peak and every valley of the original EEG signal, and the rebuilt value is almost the same. The lower figure is an EEG fragment of the onset of a seizure, where the second half of the EEG signal is quite intense and irregular. Despite this intensity and irregularity of seizure fragment, the original EEG signal can still be reconstructed from the learned features as the red line shows.

### Parameter sensitivity

In this part, we show the performance of the proposed method in various learning scenarios by tuning the number of hidden units *M* of the sparse autoencoder. In the EEG dictionary learning step, the output of the hidden layer denotes the extracted features of an EEG fragment, and each hidden unit is directly associated with each EEG word in the EEG dictionary. In practice, the number of hidden units *M* of the sparse autoencoder not only affects the training speed to a great extent, but also determines the dimensionality of the feature space in the classification step. Hence we conduct the parameter sensitivity experiment on the number of hidden units *M*.

As the input size of the sparse autoencoder is 256 *Hz*×3 *seconds*=768, the number of hidden units *M* varies in the range of 0 and 768. So we set the number of hidden units *M*=50, 150, 250, 400, 500, and measure the performance of Context-EEG respectively.

**Fig. 11:**
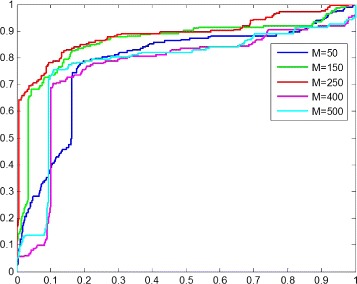
The ROC curve for the parameter sensitivity experiment. In this figure, *M* is the number of hidden units of the sparse autoencoder and we record the performances of the proposed model with *M*=50, 150, 250, 400, 500 respectively

**Fig. 12:**
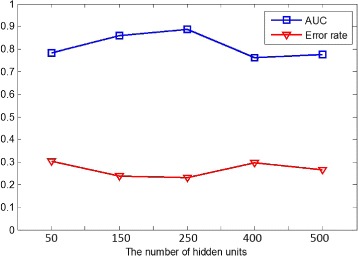
The AUC and error rate of Context-EEG with different parameter settings. In this figure, the blue line shows how the AUC of the proposed model changes as the number of hidden units gets larger and the red line denotes the error rate. The x-axis is the number of hidden units

Figure 11 shows the ROC curves with different parameter settings. The proposed model gets the best performance when *M* is 250. Comparing to the input size 768, the dimensions are reduced effectively and 250 hidden units are enough to capture the important information of the original data. While too few hidden units might result in the proposed model being unable to extract enough features, such as *M*=50, and too many hidden units might also put the proposed model at the risk of the curse of dimensionality, such as *M*=500. The AUC and error rate of parameter settings also affirms this conclusion as shown in Fig. 12. When the number of hidden units is too large or too small, the performance of Context-EEG decreases somewhat, but we can observe that it still outperforms the baseline methods.

## Conclusions

In this paper, we design and evaluate the context-learning based EEG analysis for seizure detection model (Context-EEG) that utilizes the scalp EEG to detect the onset of a seizure. The proposed model is a general, non-patient specific model which is capable of extracting both the hidden inherent features and the temporal features for the EEG signals. The hidden inherent features are extracted from each EEG fragment internally by a sparse autoencoder and the temporal features of an EEG fragment are extracted in its EEG context by the EEG context learning method. When detecting seizure with respect to a given EEG fragment, not only its internal hidden features but also the temporal features make a contribution to the classification task.

The proposed method has been tested on the CHB-MIT scalp EEG dataset and compared with several baseline methods. In general, the results show the effectiveness and superiority of the proposed model in detecting epileptic seizures. Since the proposed model is very fast at testing, once we obtain the trained model, we can detect the onset of a seizure in real time.

## Abbreviations

AUC, area under curve; AE, auto-encoder; BCI, brain-computer interface; CBOW, continuous bag of words; EEG, electroencephalogram; KL, Kullback-Leibler divergence; NN, neural network; PCA, principal component analysis; ROC, receiver operating characteristics; SAE, sparse auto-encoder; SVM, support vector machine

## References

[1] Fisher RS, Boas WVE, Blume W, Elger C, Genton P, Lee P, Engel J (2005). Epileptic seizures and epilepsy: definitions proposed by the international league against epilepsy (ilae) and the international bureau for epilepsy (ibe). Epilepsia.

[2] Wilden JA, Cohen-Gadol AA (2012). Evaluation of first nonfebrile seizures. Am Fam Phys.

[3] Berg AT (2008). Risk of recurrence after a first unprovoked seizure. Epilepsia.

[4] Clancy RR, Legido A (1991). Postnatal epilepsy after eeg-confirmed neonatal seizures. Epilepsia.

[5] Goldberger AL, Amaral LA, Glass L, Hausdorff JM, Ivanov PC, Mark RG, Mietus JE, Moody GB, Peng CK, Stanley HE (2000). Physiobank, physiotoolkit, and physionet components of a new research resource for complex physiologic signals. Circulation.

[6] Li K, Li X, Zhang Y, Zhang A (2013). Affective state recognition from eeg with deep belief networks. Bioinformatics and Biomedicine (BIBM), 2013 IEEE International Conference On.

[7] Casson AJ, Yates DC, Duncan JS, Rodriguez-Villegas E (2010). Wearable electroencephalography. Eng Med Biol Mag IEEE.

[8] Jia X, Li K, Li X, Zhang A (2014). A novel semi-supervised deep learning framework for affective state recognition on eeg signals. Bioinformatics and Bioengineering (BIBE), 2014 IEEE International Conference On.

[9] Shoeb AH. Application of machine learning to epileptic seizure onset detection and treatment. 2009. PhD thesis, Massachusetts Institute of Technology.

[10] Xun G, Li X, Knecht MR, Prasad PN, Swihart MT, Walsh TR, Zhang A (2015). Identifying inorganic material affinity classes for peptide sequences based on context learning. Bioinformatics and Biomedicine (BIBM), 2015 IEEE International Conference On.

[11] Xun G, Jia X, Zhang A (2015). Context-learning based electroencephalogram analysis for epileptic seizure detection. Bioinformatics and Biomedicine (BIBM), 2015 IEEE International Conference On.

[12] Rumelhart DE, Hinton GE, Williams RJ (1988). Learning representations by back-propagating errors. Cogn Model.

[13] Baldi P, Hornik K (1989). Neural networks and principal component analysis: Learning from examples without local minima. Neural Netw.

[14] Japkowicz N, Hanson SJ, Gluck M (2000). Nonlinear autoassociation is not equivalent to pca. Neural Comput.

[15] Olshausen BA (1996). Emergence of simple-cell receptive field properties by learning a sparse code for natural images. Nature.

[16] Kullback S, Leibler RA (1951). On information and sufficiency. Ann Math Stat..

[17] Mikolov T, Sutskever I, Chen K, Corrado GS, Dean J (2013). Distributed representations of words and phrases and their compositionality. Advances in Neural Information Processing Systems.

[18] Xun G, Yang Y, Wang L, Liu W (2012). Latent community discovery with network regularization for core actors clustering. COLING (Posters).

[19] Cortes C, Vapnik V (1995). Support-vector networks. Mach Learn.

[20] McCulloch WS, Pitts W (1943). A logical calculus of the ideas immanent in nervous activity. Bull Math Biophys.

[21] Jolliffe I. Principal Component Analysis. Wiley StatsRef: Statistics Reference Online. 2014.

